# Impact of Body Temperature in Patients With Acute Basilar Artery Occlusion: Analysis of the BASILAR Database

**DOI:** 10.3389/fneur.2022.907410

**Published:** 2022-06-03

**Authors:** Wenbin Zhang, Fengli Li, Cai Zhang, Bo Lei, Wei Deng, Hongliang Zeng, Yang Yu, Junxiong Wu, Daizhou Peng, Zhenxuan Tian, Xiurong Zhu, Zhizhou Hu, Yifan Hong, Wenbo Li, Hanming Ge, Xinwei Xu, Dongsheng Ju, Shunyu Yang, Chengde Pan, Wenjie Zi, Shouchun Wang

**Affiliations:** ^1^Department of Neurology, The First Hospital of Jilin University, Changchun, China; ^2^Department of Neurology, Xinqiao Hospital and The Second Affiliated Hospital, Army Medical University (Third Military Medical University), Chongqing, China; ^3^Department of Neurology, Daqing Oilfield General Hospital, Daqing, China; ^4^Department of Cerebrovascular Diseases, Leshan People's Hospital, Leshan, China; ^5^Department of Neurology, Xiangyang No.1 People's Hospital, Hubei University of Medicine, Xiangyang, China; ^6^Department of Neurology, Ganzhou People's Hospital, Ganzhou, China; ^7^Department of Neurology, Nanyang Central Hospital, Nanyang, China; ^8^Department of Emergency, Xiangtan Central Hospital, Xiangtan, China; ^9^Department of Neurology, Qianxinan People's Hospital, Xingyi, China; ^10^Department of Neurology, The 404th Hospital of Mianyang, Mianyang, China; ^11^Department of Neurology, Chongzhou People's Hospital, Chongzhou, China; ^12^Department of Neurology, Longyan No. 1 Hospital, Longyan, China; ^13^Department of Neurology, Shantou Central Hospital, Shantou, China; ^14^Department of Neurointervention, Luoyang Central Hospital, Luoyang, China; ^15^Department of Neurology, Xi'an Third Hospital, Xi'an, China; ^16^Department of Neurology, Jieyang People's Hospital, Jieyang, China; ^17^Department of Neurology, Songyuan Jilin Oilfield Hospital, Songyuan, China; ^18^Department of Neurology, The First People's Hospital of Yunnan Province, Kunming, China; ^19^Department of Neurology, Banan District People's Hospital, Chongqing, China

**Keywords:** basilar artery occlusion, body temperature, endovascular treatment, admission body temperature, peak body temperature

## Abstract

**Background:**

A link between body temperature and stroke outcomes has been established but not for acute basilar artery occlusion. We aimed to determine the association between body temperature and clinical outcomes in patients with acute basilar artery occlusion and temperature management range.

**Methods:**

We included patients from the Endovascular Treatment for Acute Basilar Artery Occlusion Study (BASILAR) database with records of both admission body temperature (ABT) and peak body temperature (PBT). ABT was defined as the body temperature first measured at the hospital visit, PBT was defined as the highest temperature within 24 h of treatment, and minus body temperature (MBT) was defined as PBT-ABT. The primary clinical outcome was favorable functional outcome, defined as the proportion of patients with a modified Rankin Scale score of 0–3 at 3 months. Secondary outcomes included 3-month mortality, in-hospital mortality, and symptomatic cerebral hemorrhage.

**Results:**

A total of 664 patients were enrolled in the study; 74.7% were men, with a median age of 65 (interquartile range, 57.25–74) years. In all patients, multivariate analysis indicated that PBT and MBT were independent predictors of favorable functional outcome [odds ratio (OR), 0.57 (95% CI, 0.43–0.77); OR, 0.68 (95% CI, 0.52–0.88), respectively], and higher ABT, PBT, and MBT were associated with an increased 3-month mortality [OR, 1.47 (95% CI, 1.03–2.10), OR, 1.58 (95% CI, 1.28–1.96), OR, 1.35 (95% CI, 1.11–1.65), respectively]. Proportional odds models demonstrated that when ABT, PBT, MBT were in the range of <37.5, <38.9, and −0.6–2.7°C, respectively, the benefit of the endovascular treatment is clearly greater than that of standard medical treatment in terms of favorable functional outcome.

**Conclusions:**

Body temperature is an independent predictor of clinical outcome in patients with acute basilar artery occlusion. It is necessary to control the patient body temperature within the appropriate range in clinical settings.

**Trial Registration:**

Chinese Clinical Trial Registry ChiCTR1800014759. Registered 03 February 2018. Retrospectively registered.

## Introduction

Acute ischemic stroke is the leading cause of disability worldwide and is among the leading causes of mortality (1). For large vessel occlusive strokes, reperfusion therapy is the best treatment modality. Therefore, many clinical trials have focused on symptomatology, imaging, and temporal interventions to improve the clinical prognosis of patients and guide treatment choice (2–4). Recently, few studies have focused on the prognostic impact of vital signs in patients with ischemic stroke.

The role of body temperature as an important vital sign has been simulated in stroke animal models for many years and hypothermia has been reported to be neuroprotective for brain tissue after stroke (5). However, in clinical trials, studies on the prognostic impact of body temperature on ischemic stroke are limited, and most suggest that hyperthermia leads to a poor clinical outcome (6–8). However, there is no consistent definition of hyperthermia in these studies.

Although the role of body temperature on large vessel occlusive stroke has been recognized in recent years, Diprose et al. (9) there are no reports exploring the effect of body temperature on acute basilar artery occlusive stroke. Therefore, we analyzed data from the BASILAR database to assess the effect of body temperature on the clinical outcomes of patients with acute basilar artery occlusion and aimed to find the ideal range of body temperature required.

## Methods

### Patient Selection

Our patients were selected from the Endovascular Treatment for Acute Basilar Artery Occlusion Study (BASILAR) database, a nationwide prospective cohort study including 47 comprehensive stroke centers across 15 provinces in China that aimed to assess the efficacy and safety of intervention for patients with acute BAO. Details of the study have been published previously (10). Patients with both admission body temperature (ABT) and peak body temperature (PBT) 24 h after treatment were included in this analysis.

The study is approved by Medical Ethics Committee of the Second Affiliated Hospital of Third Military Medical University of Chinese People's Liberation Army. And the approval number is 2013-087-01. In addition, the trial protocol was approved by the institutional review board at each participating hospital. Written informed consent was obtained from the patients or, if unable to provide consent, their legal surrogates, as required by the authorities' guidelines.

### Temperature and Outcome Measures

Body temperature refers to the axillary temperature measured using a mercury thermometer. Body temperature is measured at least every four hours after treatment. The ABT was defined as the body temperature measured when the patient attended the emergency department. The PBT was defined as the highest temperature within 24 h of treatment. For the endovascular treatment (EVT) group, PBT refers to 24 h after mechanical thrombectomy, and in the standard medical treatment (SMT) group, PBT refers to 24 h after intravenous thrombolysis or oral drugs. The minus body temperature (MBT) is defined as PBT-ABT.

The primary outcome was a favorable functional outcome, defined as the proportion of patients with a modified Rankin Scale (mRS) score ≤ 3 at 90 days. The secondary outcomes included in-hospital mortality, with mRS = 6 at discharge, mortality within 90 days, and symptomatic intracerebral hemorrhage (SICH) within 48 h, confirmed by brain computed tomography or magnetic resonance imaging. Intracerebral hemorrhages were evaluated according to the Heidelberg Bleeding Classification (11). SICH was defined as an increased National Institutes of Health Stroke Scale Score (NIHSS) ≥4 or any parenchymal intracerebral hemorrhage.

### Statistical Analysis

Differences between the baseline characteristics of the two groups were tested using the Mann–Whitney *U*-test for numerical variables and Pearson's Chi-square test or Fisher's exact test for categorical variables. Binary logistic regression was used to estimate the treatment effect and determine the predictors of primary and secondary outcomes. We adjusted the variables based on age, baseline NIHSS, baseline posterior circulation Alberta Stroke Program Early Computed Tomography Score (pc-ASPECTS), the Basilar Artery on Computed Tomography Angiography (BATMAN) score, type of treatment, occlusion site, diabetes mellitus, intravenous thrombolysis, systolic blood pressure, neutrophil count, leukocyte count, onset to treatment time, Trial of ORG10172 in Acute Stroke Treatment (TOAST) classification multivariate logistic regression analysis in all patients. For the EVT group, we selected age, NIHSS, pc-ASPECTS, BATMAN score, occlusion site, recanalization, and puncture to recanalization time as covariates. For the SMT group, we selected age, NIHSS, pc-ASPECTS, BATMAN score, occlusion site, intravenous thrombolysis, and onset to treatment time as covariates. In addition, to analyze how the treatment effect changed with body temperature, we constructed proportional odds models. The functional outcome at 90 days was the dependent variable in each model. Body temperature and its interaction term with the treatment modality were used as independent variables in the model. There was no interaction between any of the three body temperatures and the treatment modalities. All covariates were consistent with those in the multivariate logistic regression. In the sub-analysis, to study how the effect of recanalization and intravenous thrombolysis changed with body temperature, the same method was used again. To generate benefit curves related to the intervention modalities, outcome-specific predicted probabilities for continuous body temperature values were computed by setting other variables in the model to their mean values.

In this study, the missing values of key variables were excluded from the analysis, and thus there was no need for imputation. A tow-tailed *P* < 0.05 was considered statistically significant. Statistical analyses were performed using SPSS version 26 (IBM Corp., Armonk, NY, United States), STATA version 16 (StataCorp LLC, College Station, TX, United States). The visualized plots for proportional odds analysis were generated using RStudio software version 1.4.1717 (RStudio, PBC, Boston, MA, United States). Distribution surfaces, representing changes in predicted outcome probabilities, were generated using SigmaPlot 12.5 (Systat Software, San Jose, CA, United States), with models assessed using the *R*^2^ correlation metric.

## Results

### Baseline Characteristics

One hundred and sixty-five patients were excluded from the BASILAR database because of incomplete ABT and PBT. Thus, we analyzed 664 patients, including 518 in the EVT group and 146 in the SMT group. Among these patients, the median (interquartile range) age was 65 (57.25–74) years, baseline NIHSS was 26 (16–33) , baseline pc-ASPECTS was 8 (7-9), and BATMAN score was 4 (2-6). The median (interquartile range) ABT, PBT, and MBT were 36.6 (36.5–36.8), 37.6 (37.0–38.3), and 0.85 (0.3–1.6)°C, respectively. The rate of recanalization (modified Thrombolysis in Cerebral Infarction 2b−3) was 80.9% (419/518) in the EVT group. The rate of intravenous thrombolysis was 24.7% (36/146) in the SMT group (Table 1).

**Table 1:** Baseline, procedural, and outcome parameters.

	**All patients**	**EVT**	**SMT**	***P*** **-Value**
	**(*n* = 664)**	**(*n* = 518)**	**(*n* = 146)**	
Age, years, median, (IQR)	65 (57.25–74)	65 (57–73)	67 (59–76)	0.01
Sex male, *n* (%)	496 (74.7)	392 (75.7)	104 (71.2)	0.275
**Medical history, *n* (%)**				
Diabetes mellitus	148 (22.3)	118 (22.8)	30 (20.5)	0.567
Hypertension	469 (70.6)	362 (69.9)	107 (73.3)	0.425
Hyperlipidaemia	234 (35.2)	179 (34.6)	55 (37.7)	0.486
Smoking	224 (33.7)	191 (36.9)	33 (22.6)	0.001
Coronary artery disease	101 (15.2)	83 (16)	18 (12.3)	0.272
Atrial fibrillation	119 (17.9)	101 (19.5)	18 (12.3)	0.046
Ischemic stroke	155 (23.3)	113 (21.8)	42 (28.8)	0.079
**Index stroke data**				
Baseline NIHSS, median (IQR)	26 (16–33)	26 (16–33)	25.5 (15–32)	0.503
Baseline pc-ASPECTS, median (IQR)	8 (7–9)	8 (7–9)	7 (6–8)	<0.001
BATMAN score, median (IQR)	4 (2–6)	4 (2–5)	5 (3–6)	0.001
SBP, mm Hg, median (IQR)	152 (136–170)	150 (135–166.5)	156.5 (141.75–174.25)	0.003
**Body temperature, infection index, median (IQR)**				
ABT, °C	36.6 (36.5–36.8)	36.6 (36.5–36.8)	36.7 (36.5–36.9)	0.037
PBT, °C	37.6 (37–38.3)	37.5 (37–38.225)	37.7 (37.2–38.5)	0.004
MBT, °C	0.85 (0.3–1.6)	0.8 (0.3–1.5)	1 (0.38–1.7)	0.165
WBC, × 10^9^/L	11.1 (8.43–13.79)	11.18 (8.39–13.79)	10.94 (8.96–13.8)	0.873
Neutrophil, × 10^9^/L	9.25 (6.63–12.02)	9.29 (6.6–12.02)	9 (6.81–12.09)	0.728
Lymphocyte, × 10^9^/L	1.15 (0.8–1.6)	1.14 (0.8–1.6)	1.16 (0.83–1.57)	0.862
CRP, nmol/L	77.62 (32.67–218.57)	70.48 (31.90–189.52)	126.67 (43.81–387.62)	0.035
PCT, μg/L	0.1 (0.05–0.31)	0.1 (0.05–0.33)	0.08 (0.05–0.21)	0.195
**Occlusion sites *n* (%)**				
Distal basilar artery	213 (32.1)	176 (34)	37 (25.3)	<0.001
Middle basilar artery	237 (35.7)	156 (30.1)	81 (55.5)	
Proximal basilar artery	102 (15.4)	90 (17.4)	12 (8.2)	
Vertebral artery-V4 artery	112 (16.9)	96 (18.5)	16 (11)	
**Stroke causative mechanism *n* (%)**				
Large artery atherosclerosis	438 (66)	343 (66.2)	95 (65.1)	0.005
Cardioembolism	158 (23.8)	132 (25.5)	26 (17.8)	
Other	18 (2.7)	14 (2.7)	4 (2.7)	
Unknown	50 (7.5)	29 (5.6)	21 (14.4)	
**Treatment profiles**				
Intravenous thrombolysis, *n* (%)	122 (18.4)	86 (16.6)	36 (24.7)	0.026
Recanalization, *n* (%)	NA	419 (80.9)	NA	NA
Onset to treatment time, min, median (IQR)	250.5 (132.25–406)	252.5 (137.75–403.75)	240 (118.5–412.25)	0.691
Onset to recanalization time, min, median (IQR)	NA	453 (337–645)	NA	NA
Puncture to recanalization time, min, median (IQR)	NA	106 (71–155)	NA	NA
LOS, days (IQR)	11 (3–20)	12 (4–20)	6 (2–14.5)	<0.001

*IQR, indicated interquartile range; NIHSS, National Institutes of Health Stroke Scale; pc-ASPECTS, posterior circulation Alberta Stroke Program Early Computed Tomography Score; BATMAN, the Basilar Artery on Computed Tomography Angiography; ABT, admission body temperature; PBT, peak body temperature; MBT, minus of body temperature; WBC, white blood cell; CRP, C-reactive protein; PCT, procalcitonin; LOS, length of stay; NA, not applicable*.

### Association Between Temperature and Outcomes in BAO Patients

Multivariate logistic regression adjusted for potential confounders demonstrated that PBT and MBT were independent predictors of favorable functional independence [OR, 0.57 (95% CI, 0.43–0.77), *P* <0.001; OR, 0.68 (95% CI, 0.52–0.88), *P* = 0.004, respectively]. ABT, PBT, and MBT were independent predictors of 90-day mortality [odds ratio (OR), 1.47 (95% CI, 1.03–2.10), *P* = 0.034; OR, 1.58 (95% CI, 1.28–1.96), *P* < 0.001; OR, 1.35 (95% CI, 1.11–1.65), *P* = 0.003, respectively]. Furthermore, PBT was an independent predictor of SICH [OR, 1.77 (95% CI, 1.21–2.56), *P* = 0.003] (Table 2). For the three different categories of body temperature, other independent predictors are shown in the Supplementary Tables I–III.

**Table 2:** Multivariate analysis: association between body temperature and outcomes.

	**ALL (*n* = 664)**				**EVT (*n* = 518)**				**SMT (*n* = 146)**			
	**Unadjusted OR** **(95% CI%)**	***P*** **-Value**	**Adjusted OR (95% CI)**	***P*** **-Value**	**Unadjusted OR** **(95% CI%)**	***P*** **-Value**	**Adjusted OR (95% CI)**	***P*** **-Value**	**Unadjusted OR** **(95% CI%)**	***P*** **-Value**	**Adjusted OR (95% CI)**	***P*** **-Value**
**ABT**												
3-month mRS 0–3	0.47 (0.31–0.69)	<0.001	0.70 (0.44–1.12)	0.139	0.44 (0.29–0.68)	<0.001	0.57 (0.33–0.97)	0.039	0.94 (0.38–2.32)	0.897	1.37 (0.43–4.37)	0.591
3-month mortality	1.95 (1.43–2.65)	<0.001	1.47 (1.03–2.10)	0.034	2.03 (1.43–2.89)	<0.001	1.63 (1.08–2.45)	0.019	1.38 (0.71–2.65)	0.341	1.05 (0.48–2.30)	0.897
In-hospital mortality	1.26 (0.95–1.66)	0.105	0.93 (0.67–1.29)	0.677	1.30 (0.93–1.82)	0.119	1.02 (0.71–1.47)	0.927	1.04 (0.63–1.73)	0.875	1.21 (0.66–2.19)	0.541
SICH	1.53 (0.99–2.38)	0.057	1.51 (0.90–2.54)	0.120	1.64 (11.03–2.61)	0.036	1.43 (0.86–2.36)	0.165	1.72 (0.26–11.59)	0.579	^†^	^†^
**PBT**												
3-month mRS 0–3	0.45 (0.36–0.57)	<0.001	0.57 (0.43–0.77)	<0.001	0.47 (0.37–0.60)	<0.001	0.56 (0.41–0.75)	<0.001	0.46 (0.20–1.04)	0.061	0.34 (0.12–0.93)	0.036
3-month mortality	1.95 (1.63–2.35)	<0.001	1.58 (1.28–1.96)	<0.001	1.82 (1.48–2.23)	<0.001	1.46 (1.15–1.87)	0.002	2.47 (1.45–4.22)	0.001	2.40 (1.30–4.45)	0.005
In-hospital mortality	1.38 (1.16–1.64)	<0.001	1.20 (0.97–1.48)	0.088	1.32 (1.07–1.62)	0.008	0.97 (0.54–1.73)	0.915	1.43 (1.02–2.02)	0.041	1.74 (1.12–2.70)	0.014
SICH	1.64 (1.21–2.21)	0.001	1.77 (1.21–2.59)	0.003	1.73 (1.26–2.37)	0.001	1.50 (1.06–2.13)	0.022	2.36 (0.62–9.00)	0.210	^†^	^†^
**MBT**												
3-month mRS 0–3	0.62 (0.51–0.77)	<0.001	0.68 (0.52–0.88)	0.004	0.65 (0.52–0.81)	<0.001	0.68 (0.52–0.90)	0.007	0.46 (0.20–1.07)	0.071	0.41 (0.15–1.11)	0.078
3-month mortality	1.49 (1.26–1.77)	<0.001	1.35 (1.11–1.65)	0.003	1.40 (1.16–1.70)	0.001	1.20 (0.96–1.51)	0.112	2.30 (1.23–3.35)	0.006	2.14 (1.20–3.82)	0.01
In-hospital mortality	1.28 (1.07–1.52)	0.007	1.23 (1.00–1.51)	0.053	1.21 (0.98–1.49)	0.082	1.00 (0.80–1.26)	0.98	1.41 (1.00–1.98)	0.052	1.45 (0.97–2.17)	0.071
SICH	1.41 (1.02–1.94)	0.036	1.44 (0.99–2.09)	0.056	1.46 (1.04–2.05)	0.029	1.27 (0.89–1.81)	0.18	1.88 (0.51–6.96)	0.347	^†^	^†^

*OR, odds ratio; CI, confidence interval; ABT, admission body temperature; PBT, peak body temperature; MBT, minus body temperature; mRS, modified Rankin Scale; NA, not applicable;*

† *Not enough data available to calculate statistical parameter*.

**Figure 1:**
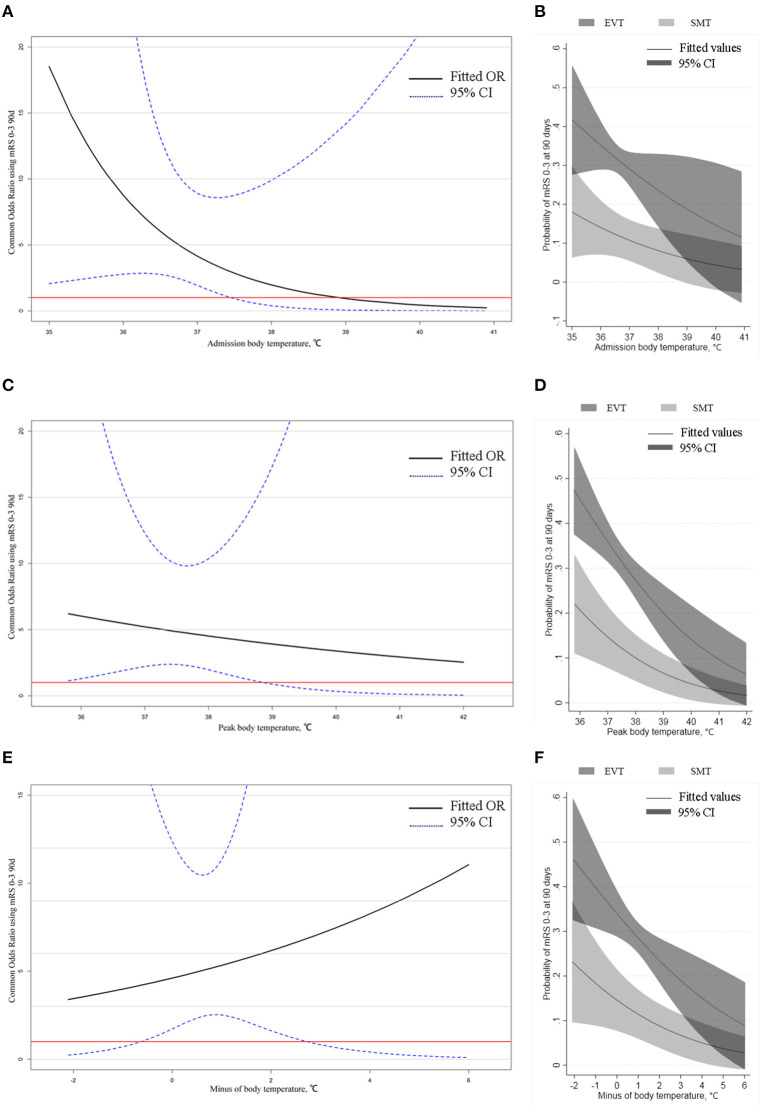
Odds ratio mRS 0–3 90 days in EVT vs. SMT by body temperature in all patients and predicted probability of favorable outcome by body temperature. **(A,C,E)** Odds ratio mRS 0–3 90 days in EVT vs. SMT by admission body temperature, peak body temperature, and minus body temperature. In all images, the threshold indicates the intersection of the lower limit of the confidence interval with the red line, and the part above the threshold indicates that the benefit of the EVT group is clearly greater than that of the SMT. **(B,D,F)** Curves showed that the probability of a favorable outcome decreases with increasing body temperature in all three categories, and the probability of a favorable outcome is determined to be higher in the EVT group than in the SMT group in the corresponding temperature range, consistent with the results of **(A,C,E)**.

The proportional odds models demonstrated that when ABT was <37.5°C, PBT <38.9°C, and MBT−0.6–2.7°C, there was a clear benefit in favorable functional outcome for the EVT group compared to the SMT group (Figures 1A,C,E).

### Association Between Temperature and Outcomes in the Subgroup Analysis

In the EVT group, multivariate logistic regression adjusted for potential confounders demonstrated that ABT, PBT, and MBT were independent predictors of favorable functional independence [OR, 0.57 (95% CI, 0.33–0.97), *P* = 0.039; OR, 0.56 (95% CI, 0.41–0.75), *P* < 0.001; OR, 0.68 (95% CI, 0.52–0.90), *P* = 0.007, respectively]. For 90-day mortality, ABT and PBT were independent predictors [OR, 1.68 (95% CI, 1.08–2.45), *P* = 0.019; OR, 1.46 (95% CI, 1.15–1.87), *P* = 0.002, respectively]. Moreover, PBT was also an independent predictor of SICH [OR, 1.50 (95% CI, 1.06–2.13), *P* = 0.022] (Table 2). For the three different categories of body temperature, other independent predictors are shown in the Supplementary Tables I–III.

**Figure 2:**
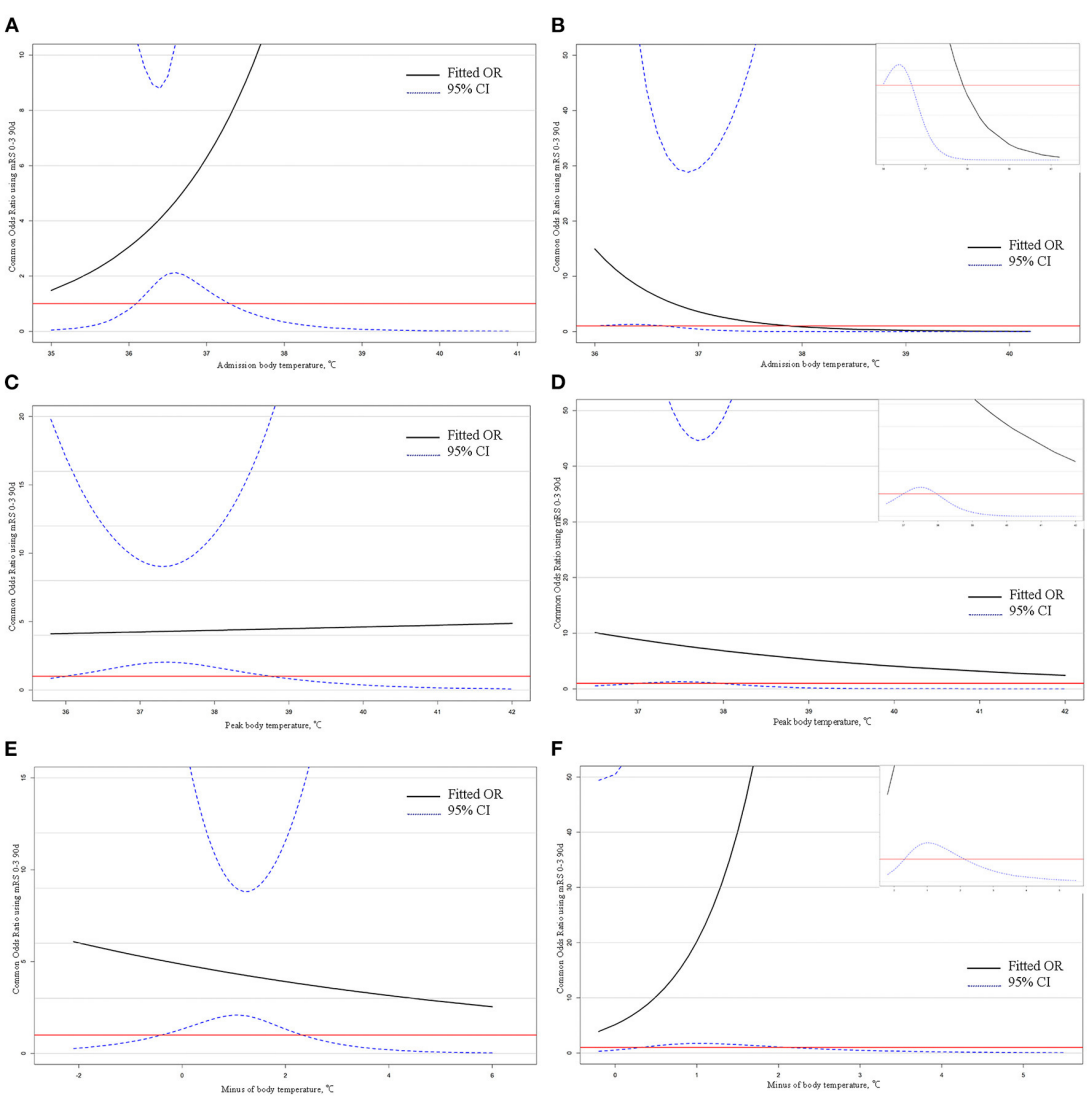
Odds ratio mRS 0–3 90 days in recanalization vs. non-recanalization and intravenous thrombolysis vs. no intravenous thrombolysis by body temperature in subgroup. **(A,C,E)** Odds ratio mRS 0–3 90 days in recanalization vs. non-recanalization by admission body temperature, peak body temperature, and minus body temperature. In all images, the threshold indicates the intersection of the lower limit of the confidence interval with the red line, and the part above the threshold indicates that the benefit of the recanalization group is clearly greater than that of the non-recanalization. **(B,D,F)** Odds ratio mRS 0–3 90 days in intravenous thrombolysis vs. no intravenous thrombolysis by admission body temperature, peak body temperature, and minus body temperature. The upper right corner of the images indicates a partial zoom. In all images, the threshold indicates the intersection of the lower limit of the confidence interval with the red line, and the part above the threshold indicates that the benefit of the intravenous thrombolysis group is clearly greater than that of the no intravenous thrombolysis.

The proportional odds models illustrated that when ABT is in the range of 36.1–37.3°C, PBT is in the range of 36–38.8°C, and MBT is in the range of −0.4–2.3°C, there is a clear benefit to recanalization relative to non-recanalization in the EVT group (Figures 2A,C,E).

In the SMT group, ABT was no longer an independent factor affecting any outcomes, and PBT was a predictor of favorable functional outcome, in-hospital mortality and 90-day mortality [OR, 0.34 (95% CI, 0.12–0.93), *P* = 0.036; OR, 1.737 (95% CI, 1.12–2.70), *P* = 0.014; and OR, 2.40 (95% CI, 1.30–4.45), *P* = 0.005, respectively]. MBT was an independent predictor of SICH [OR, 2.14 (95% CI, 1.20–3.82), *P* = 0.01] (Table 2). For the three different categories of body temperature, other independent predictors are shown in the Supplementary Tables I–III.

The proportional odds models showed that there is a clear benefit to intravenous thrombolysis relative to no intravenous thrombolysis in the SMT group when ABT is in the range of 36.0–36.7°C, PBT is in the range of 37.0–38.0°C, and MBT is in the range of 0.3–2.1°C (Figures 2B,D,F).

Figures 1B,D,F illustrates the predicted probabilities of favorable functional outcomes of both treatment modalities with the three different categories of body temperature as continuous variables. As body temperature increased in all three categories, the probabilities of favorable functional outcomes progressively diminished in both the EVT and SMT groups. In the subgroup analysis, the predicted probabilities of favorable functional outcomes with or without recanalization and with or without intravenous thrombolysis, the three types of body temperature as variables are presented in Supplementary eFigures I, II.

### Outcome of All Patients Associated With Temperature and Age

**Figure 3:**
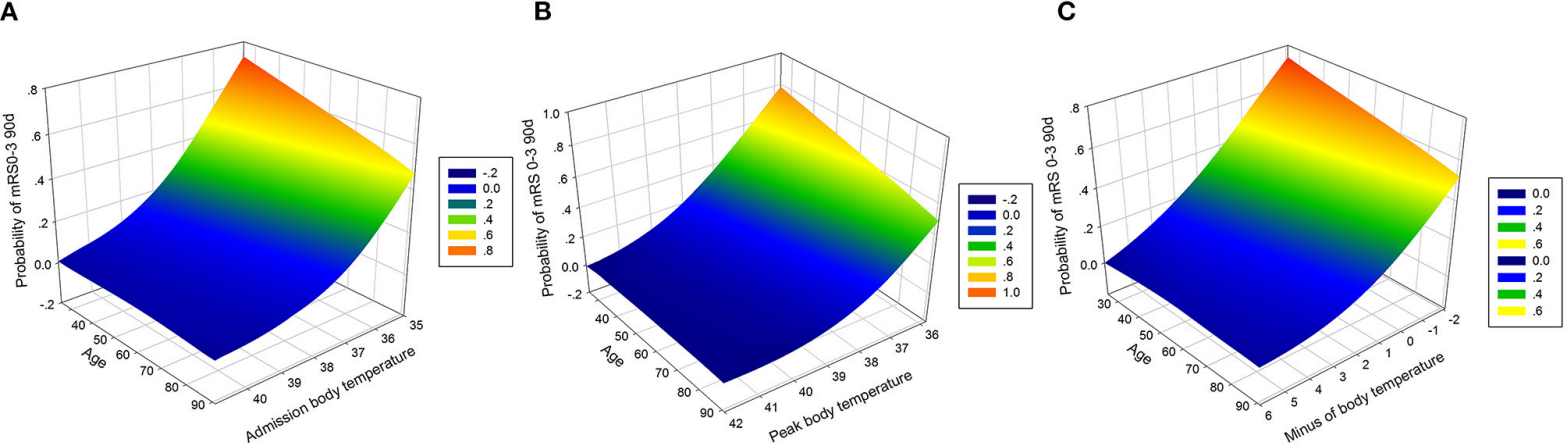
Association of body temperature and age with favorable outcome. **(A)** Association of admission body temperature and age with the probability of a favorable outcome. Favorable outcome probabilities are stable across ages in a prescribed admission body temperature (*R*^2^ = 0.9984). **(B)** Association of peak body temperature and age with the probability of a favorable outcome. Favorable outcome probabilities are stable across ages in a prescribed peak body temperature (*R*^2^ = 0.9972). **(C)** Association of minus body temperature and age with the probability of a favorable outcome. Favorable outcome probabilities are stable across ages in a prescribed minus body temperature (*R*^2^ = 0.9976).

The probabilities of favorable functional outcome decreased with age when ABP, PBT, and MBT were at low levels, while the probabilities of favorable functional outcome did not change significantly with age or did not change when ABT, PBT, and MBT were at high levels (Figure 3).

### Factors Related to Body Temperature

Supplementary eFigure III shows the correlations between body temperature and other variables. Although there were correlations between body temperature and some variables such as white blood cell count, neutrophil count, onset to door time, onset to recanalization time etc., the correlations were generally weak, with *r* values <0.3 (Supplementary Table IV).

## Discussion

This study, to the best of our knowledge, is the first to examine the relationship between body temperature and the outcomes of the posterior circulation large vessel-acute basilar artery occlusion, where PBT and MBT were independent predictors of favorable functional outcome so that for every 1°C increase in temperature, there was a 43% and 32% decrease in favorable functional outcomes, respectively. Additionally, we found specific ranges of body temperature to guide temperature management, which favored a better outcome. The benefits for the EVT group were clearly greater than those of the SMT group when the ABT was <36.5°C. To ensure that the benefit of the EVT group is better, PBT and MBT should be maintained at <38.9°C and between −0.6 and 2.7°C, respectively.

In animal studies, the outcome of local or global ischemia in brain tissue is profoundly influenced by changes in body temperature (5). Possible mechanisms of action for this effect include the following: enhanced release of neurotransmitters; exaggerated oxygen radical production; more extensive blood-brain barrier breakdown; increased numbers of potentially damaging ischemic depolarizations in the focal ischemic penumbra; impaired recovery of energy metabolism and enhanced inhibition of protein kinases; and worsening of cytoskeletal proteolysis (12). In clinical studies, a correlation between the increase in cerebral infarction and body temperature after stroke has also been reported (13–15). It is reasonable to assume that there is an association between body temperature and outcomes in patients with acute BAO.

Our study showed that ABT was not associated with a favorable functional outcome in all patients with BAO, which is consistent with previous studies (8, 16, 17). Wang et al. (18) analyzed 437 patients with ischemic stroke and found that admission temperature was an independent clinical predictor of stroke mortality, and hypothermia was associated with reduced in-hospital mortality. We did not find a statistically significant association between ABT and in-hospital mortality, but ABT was an independent predictor of 90-day mortality. We postulate that this difference may be related to differences between patients in the two studies. In the study by Wang et al. patients had milder general symptoms and 76.9% of patients were conscious. In our study, median baseline NIHHS for patients with acute basilar artery occlusion was 26, suggesting that symptoms were more severe than those in the aforementioned study. One study reported that a high ABT predicted a poor prognosis (6). Our results indicate that for patients with acute BAO with an ABT <37.5°C, a temperature that coincides with the high-temperature cut-off point of previous studies, (13, 16, 18) the benefit of EVT is significantly better than that of SMT, which is clinically relevant.

A small study (patients, *n* = 44) found that the highest temperature in patients with stroke occurs 1.5–2 days after an ischemic stroke (19). However, a study published by Geurts et al. (14) including 419 patients, reported that the highest body temperature was found on the first day after ischemic stroke. Considering that patients with posterior circulation stroke may be more susceptible to ischemic damage to the hypothalamus and that patients with basilar artery occlusion tend to be more symptomatic, the improvement of symptoms in the first 24 h has a significant predictive and clinical value. The earlier the intervention to alter the temperature, the greater the possible benefit. Furthermore, as the length of hospital stay increases, interference factors affecting temperature may introduce a significant error in our study. Therefore, we chose the highest body temperature in the first 24 h of treatment as the focus of our study.

We have shown that PBT is associated with poor outcomes, which is consistent with the results of previous studies (9, 20). A large retrospective study indicated that in the first 24 h following intensive care unit admission, a PBT ≥39°C or <37°C increased the risk of in-hospital mortality (7). Although we demonstrated that the benefit of the PBT <38.9°C in the EVT group was greater than that in the SMT group, PBT was not an independent risk factor of in-hospital mortality. We consider that there are two main reasons for this: first, the study included a relatively small number of cases and second, the patients in this study had strokes caused by acute basilar artery occlusion. When we performed univariate logistic regression, PBT was correlated with in-hospital mortality, although when we adjusted the variables based on occlusion site, treatment modality, and baseline NIHSS, etc. the *P*-value for PBT changed to 0.081, indicating that for patients with acute BAO in-hospital mortality was primarily determined by other factors and that PBT did not independently predict in-hospital mortality. In addition, only PBT was an independent predictor of SICH among the three categories of body temperature, which may be related to the mechanism by which high temperatures exacerbate blood-brain barrier disruption.

The MBT is a variable that has not been previously studied. Since MBT had a strong linear association with PBT (*r* = 0.802), PBT was also an independent predictor of the corresponding outcomes if MBT was an independent predictor of outcomes. It may appear that the variable MBT is redundant. The clinical significance of MBT indicates the tolerance of temperature variation, with a clearer benefit in the EVT group when the temperature variation is between −0.6 and 2.7°C for patients with acute basilar artery occlusion. This complements the clinical role of PBT and ABT, and a combination of the three is necessary for better temperature management.

Successful recanalization is an important prognostic predictor of endovascular treatment (21). However, not all patients who are successfully recanalized have favorable functional outcomes. Successful recanalization with a poor outcome is called futile recanalization (22). A meta-analysis of five randomized controlled trials of emergency embolization suggests a 54% incidence of futile recanalization of the anterior circulation (21). Another study that included 165 cases suggests a higher incidence of futile recanalization in the posterior circulation compared to the anterior circulation (23). Our futile recanalization rate of 63.2% (265/419) is essentially the same as the rate of 62.8% in the BASILAR study and validates the findings that the incidence of posterior circulation futile recanalization is higher than that of the anterior circulation. A 2020 study examining the prognostic impact of intra-ischemic and post-ischemic body temperatures in large vessel occlusion indicates that pre-and post-EVT body temperatures predict clinical outcomes in patients with large vessel occlusion, supported by our findings, Diprose et al. (9) despite the former study including only 17.4% (75/432) patients with posterior circulation stroke. To reduce the occurrence of futile recanalization and ensure the maximum benefit for patients, we recommend controlling ABT, PBT, and MBT in the appropriate ranges.

In a 2015 study on the association between body temperature and recanalization of acute internal carotid artery occlusion, it was noted that recanalization was not correlated with body temperature and thrombolysis (24). However, a 2010 study suggests that high admission temperature is associated with good short-term clinical outcomes in post-thrombolytic stroke patients (25). In contrast, Ernon et al. (6) report that high temperature relative to baseline body temperature within 24 h after thrombolysis was associated with poor prognosis in 2006. The same thrombolysis was performed after stroke, and three different associations between body temperature and outcome were observed. The main reason is that the former study was on stroke patients with large vessel occlusion; therefore, ABT was not associated with prognosis, which is consistent with the results of our study; a high ABT was associated with short-term good clinical outcomes in the middle study, which may be attributed to the inclusion of patients with milder symptoms (median NIHSS = 10); the results of the last study are consistent with those of our study when considering findings in relation to PBT. Furthermore, we hypothesize that in-hospital mortality is strongly associated with PBT. Only in the SMT group was there an association between in-hospital mortality and body temperature, which, in our view, should be attributed to the less pronounced improvement in symptoms and more rapid deterioration in the SMT group. Although, in most cases, intravenous thrombolysis did not have an independent influence on prognosis in the SMT group, we still found a temperature range in which thrombolysis was superior to non-thrombolysis, which is also necessary for the SMT group in clinical setting.

Temperature is a predictor of clinical outcome in stroke, and a higher temperature indicates poorer clinical prognosis (9). However, a lower temperature is not necessarily better. Several studies have shown that hypothermia is not more effective than maintaining normothermia (26, 27). For acute ischemic stroke, hypothermia is not a substitute for reperfusion therapy. Two large randomized controlled studies were terminated due to the ineffectiveness of hypothermia (28, 29). Temperature management is an important step for good prognosis after reperfusion therapy. We recommend that the three body temperatures be controlled within the appropriate range, but further research is needed on how to manage the temperature.

Our study has some limitations. First, as it was an observational study, there may be biases. Second, we did not include those patients who lacked ABT or PBT, which may be biases. Third, Axillary temperature measurements are not sufficient to reflect a patient's core temperature compared to tympanic or bladder measurements. We may be underestimating the actual body temperature of the patients. Fourth, the infection status of patients before admission and whether they were taking antipyretic drugs such as ibuprofen were unknown. Inflammatory complications such as pneumonia may have occurred during hospitalization after 24 h of treatment. Thus, we did not include pre-hospital and post-hospital medication, treatment, and infection, which may lead to discrepancies between our results and those in the real world. Fifth, although we adjusted for confounding factors in the multivariate analysis, unmeasured residual confounding factors may still exist. Finally, patient prognosis is thought to be related to prolonged temperature effects. We only investigated the prognostic impact of ABT and PBT within 24 h of treatment, a limitation that may lead to inadequate management of patient temperatures during hospitalization.

## Conclusions

Body temperature is an independent predictor of clinical outcomes in patients with acute basilar artery occlusion, and it is necessary to control the body temperature within the appropriate range in clinical settings.

## Data Availability Statement

The raw data supporting the conclusions of this article will be made available by the authors, without undue reservation.

## Ethics Statement

The studies involving human participants were reviewed and approved by Medical Ethics Committee of the Second Affiliated Hospital of Third Military Medical University of Chinese People's Liberation Army. The patients/participants provided their written informed consent to participate in this study.

## Author Contributions

SW and WZi contributed to the study conception or design and the acquisition, analysis, and interpretation of data. WZh, FL, and CZ contributed to the study conception or design and the interpretation of data. BL, WD, HZ, YY, JW, DP, ZT, XZ, ZH, YH, WL, HG, XX, DJ, SY, and CP contributed to the analysis and/or interpretation of data. All authors revised the work critically for important intellectual content and provided final approval of the version to be published.

## Funding

The authors disclose receipt of the following financial support for the research, authorship, and/or publication of this article: this work was supported by the National Natural Science Foundation of China (No. 82071323), Chongqing Natural Science Foundation (cstc2020jcyj-msxmX0926), Chongqing Science and Health Joint Project (No. 2019ZDXM002), the Army Medical University Clinical Medical Research Talent Training Program (Nos. 2018XLC3039, 2019XLC2008, and 2019XLC3016), and Jilin Province Health Talent Special Project (JLSCZD2019-013).

## Conflict of Interest

The authors declare that the research was conducted in the absence of any commercial or financial relationships that could be construed as a potential conflict of interest.

## Publisher's Note

All claims expressed in this article are solely those of the authors and do not necessarily represent those of their affiliated organizations, or those of the publisher, the editors and the reviewers. Any product that may be evaluated in this article, or claim that may be made by its manufacturer, is not guaranteed or endorsed by the publisher.
