# Supplementary material for: Impact of Body Temperature in Patients With Acute Basilar Artery Occlusion: Analysis of the BASILAR Database
Source: Front Neurol. 2022 Jun 3;13:907410. doi: 10.3389/fneur.2022.907410 (PMC9205153; doi:10.3389/fneur.2022.907410)
Supplement: Supplementary file 1 [file Table_1.DOCX]

**Supplementary Materials**

**Contents**

**eFigureⅠPredicted probability of favorable outcome by body temperature** 1

**eFigureⅡPredicted probability of favorable outcome by body temperature** 2

**eFigure Ⅲ Matrix Scatter Plot : Correlation of body temperature with other variables** 3

**Table Ⅰ Multivariable Logistic Regression Model of Outcome Measures by ABT** 4

**Table Ⅱ Multivariable Logistic Regression Model of Outcome Measures by PBT** 6

**Table Ⅲ Multivariable Logistic Regression Model of Outcome Measures by MBT** 8

**Table Ⅳ Correlation of body temperature with other variables** 10

**eFigureⅠPredicted probability of favorable outcome by body temperature**

**
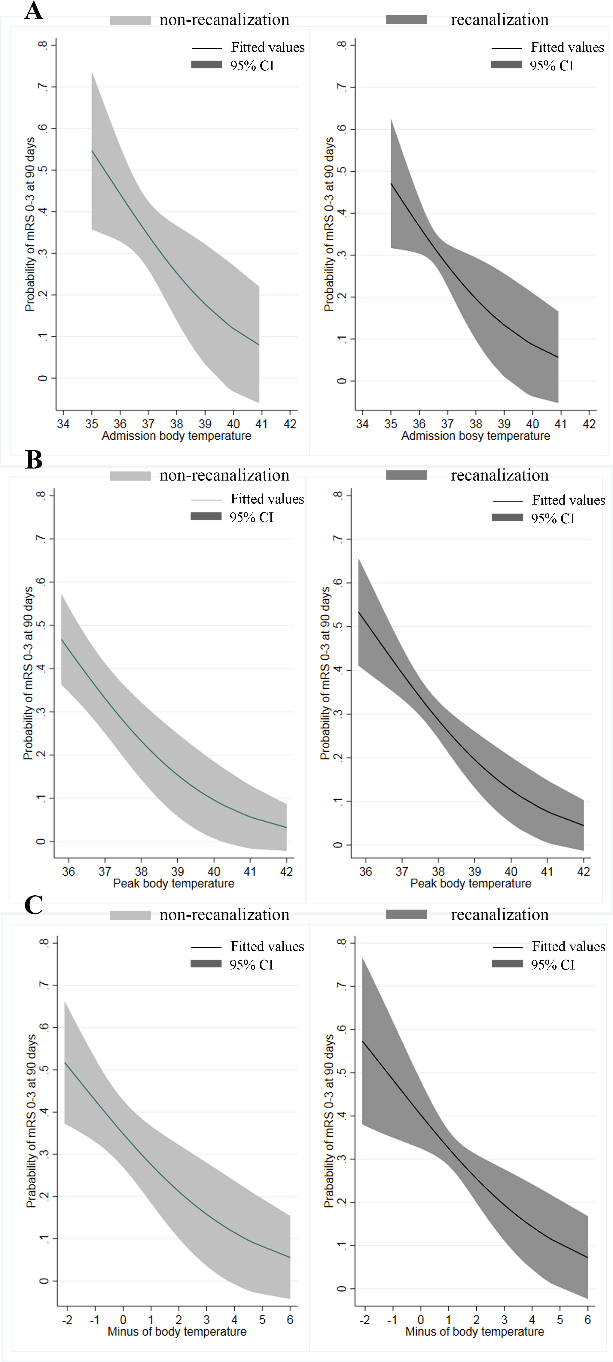
**

Curves showed that the probability of a favorable outcome decreases with increasing body temperature in all three categories.

**eFigureⅡPredicted probability of favorable outcome by body temperature**

**
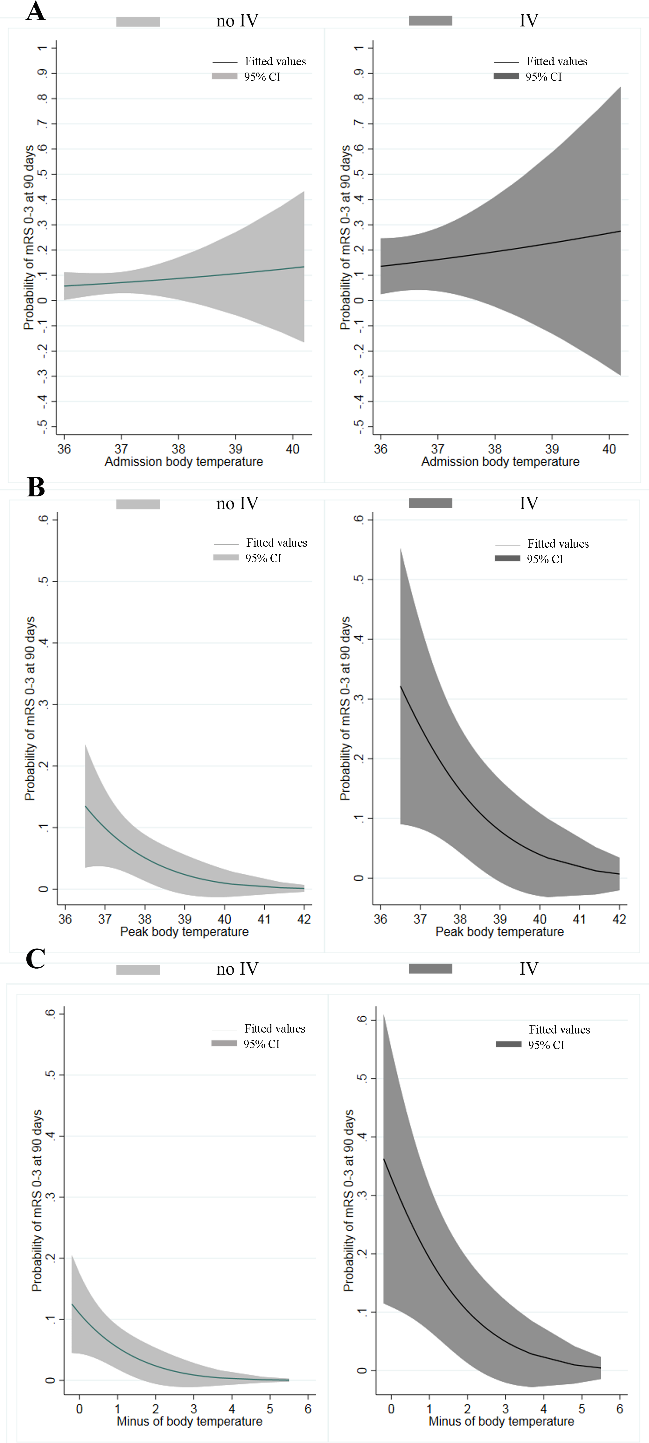
**

Curves showed that the probability of a favorable outcome decreases with increasing in peak body temperature and minus body temperature. And the probability of a favorable outcome increases with increasing in admission body temperature lightly.

**eFigure Ⅲ Matrix Scatter Plot : Correlation of body temperature with other variables**


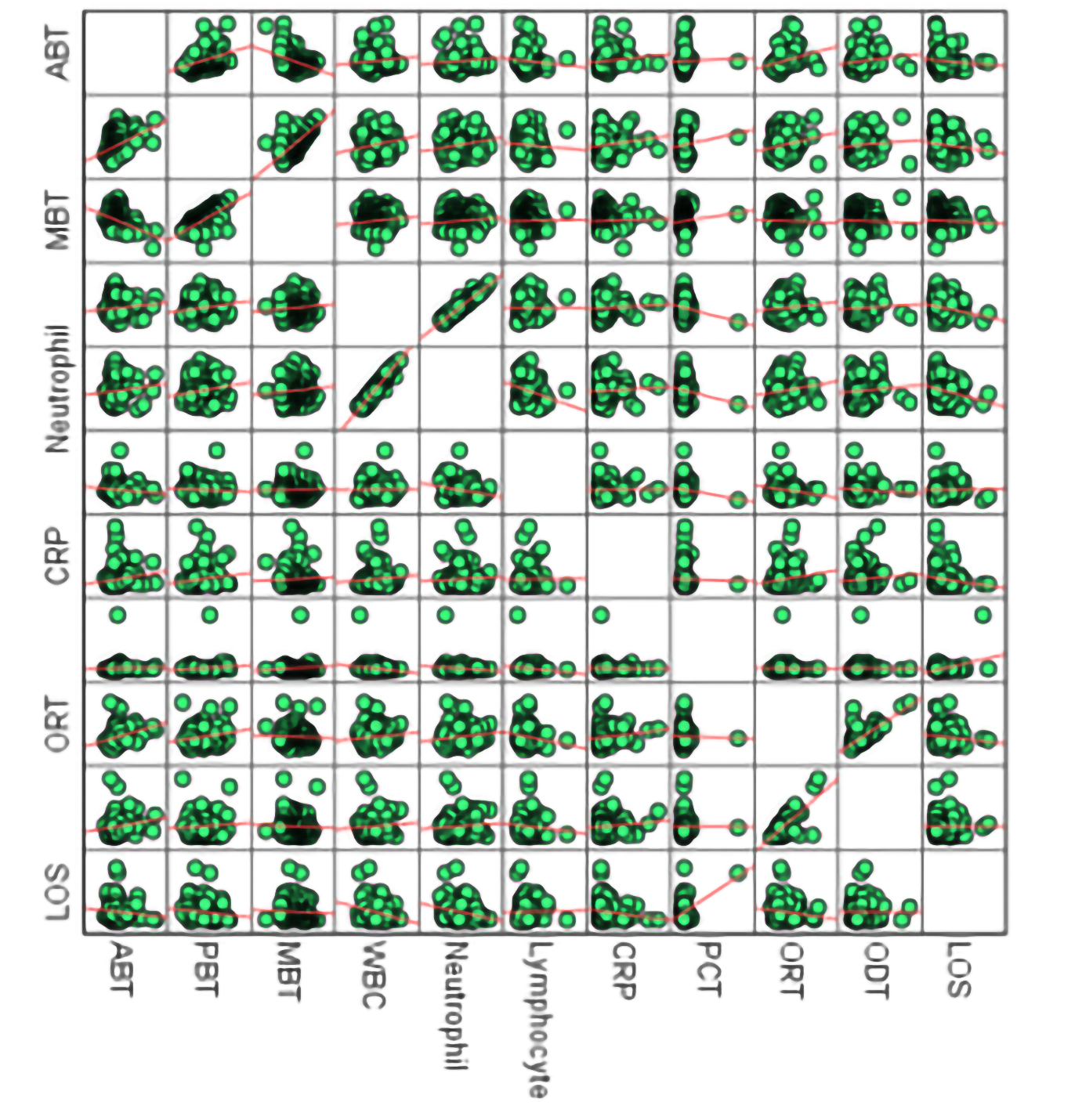
Abbreviations: ABT, admission body temperature; PBT, peak body temperature; MBT, minus of body temperature; WBC, white blood cell; CRP, C–reactive protein; PCT, procalcitonin; ODT, onset to door time; ORT, onset to recanalization time; LOS, length of stay.

The body temperatures don’t have significant link with other variables.

**Table Ⅰ Multivariable Logistic Regression Model of Outcome Measures by ABT**

| ALL | Adjusted OR (95% CI) | *P* Value | EVT | Adjusted OR (95% CI) | *P* Value | SMT | Adjusted OR (95% CI) | *P* Value |
| --- | --- | --- | --- | --- | --- | --- | --- | --- |
| 3-month mRS 0-3 | | | | | | | | |
| NIHSS | 0.93(0.91–0.95) | <0.001 | NIHSS | 0.92(0.89–0.94) | <0.001 | NIHSS | 0.91(0.85–0.99) | 0.024 |
| pc–ASPECTS | 1.68(1.43–1.97) | <0.001 | pc–ASPECTS | 1.65(1.40–1.94) | <0.001 | pc–ASPECTS | 1.79(1.02–3.13) | 0.041 |
| group | 4.54(2.15–9.59) | <0.001 | NA | NA | NA | NA | NA | NA |
| Occlusion site |  |  | Occlusion site |  |  | NA | NA | NA |
| Distal basilar artery | reference | 0.001 | Distal basilar artery | reference | 0.012 | NA | NA | NA |
| Middle basilar artery | 0.28(0.15–0.51) | <0.001 | Middle basilar artery | 0.42(0.23–0.76) | 0.004 | NA | NA | NA |
| Proximal basilar artery | 0.57(0.28–1.16) | 0.12 | Proximal basilar artery | 0.75(0.38–1.48) | 0.409 | NA | NA | NA |
| Vertebral artery–V4 segment | 0.43(0.21–0.90) | 0.025 | Vertebral artery–V4 segment | 0.42(0.21–0.83) | 0.012 | NA | NA | NA |
| NA | NA | NA | PRT | 0.99(1.00–1.00) | 0.001 | NA | NA | NA |
| NA | NA | NA | Recanalization | 4.23(2.03–8.91) | <0.001 | NA | NA | NA |
| 3-month mortality | | | | | | | | |
| Age | 1.03(1.01–1.05) | 0.002 | Age | 1.02(1.00–1.04) | 0.017 | NIHSS | 1.05(1.01–1.10) | 0.021 |
| NIHSS | 1.06(1.04–1.08) | <0.001 | NIHSS | 1.08(1.05–1.11) | <0.001 | BATMAN | 0.69(0.54–0.89) | 0.004 |
| pc–ASPECTS | 0.78(0.69–0.88) | <0.001 | pc–ASPECTS | 0.76(0.67–0.87) | <0.001 | NA | NA | NA |
| BATMAN | 0.88(0.80–0.98) | 0.015 | PRT | 1.01 (1.00–1.01) | <0.001 | NA | NA | NA |
| group | 0.28(0.17–0.46) | <0.001 | Recanalization | 0.13(0.07–0.24) | <0.001 | NA | NA | NA |
| SBP | 1.01(1.00–1.02) | 0.04 | NA | NA | NA | NA | NA | NA |
| Occlusion site |  |  | NA | NA | NA | NA | NA | NA |
| Distal basilar artery | reference | 0.192 | NA | NA | NA | NA | NA | NA |
| Middle basilar artery | 1.80(1.05–3.06) | 0.031 | NA | NA | NA | NA | NA | NA |
| Proximal basilar artery | 1.34(0.71–2.54) | 0.369 | NA | NA | NA | NA | NA | NA |
| Vertebral artery–V4 segment | 1.44(0.77–2.67) | 0.252 | NA | NA | NA | NA | NA | NA |
| In–hospital mortality 90d | | | | | | | | |
| Age | 1.02(1.00–1.04) | 0.049 | NIHSS | 1.07(1.04–1.10) | <0.001 | NIHSS | 1.05(1.01–1.10) | 0.016 |
| NIHSS | 1.06(1.03–1.08) | <0.001 | BATMAN | 0.85(0.75–0.97) | 0.013 | pc–ASPECTS | 0.73(0.58–0.93) | 0.009 |
| pc–ASPECTS | 0.86(0.77–0.97) | 0.013 | PRT | 1.00(1.00–1.01) | 0.013 | Occlusion site |  |  |
| Neutrophils | 1.29(1.02–1.62) | 0.032 | Recanalization | 0.42(0.25–0.70) | 0.001 | Distal basilar artery | reference | 0.22 |
| group | 0.43(0.26–0.70) | 0.001 | NA | NA | NA | Middle basilar artery | 3.07(1.04–9.08) | 0.43 |
| Diabetes mellitus | 1.80(1.14–2.84) | 0.011 | NA | NA | NA | Proximal basilar artery | 1.97(0.32–11.98) | 0.466 |
| NA | NA | NA | NA | NA | NA | Vertebral artery–V4 segment | 2.90(0.70–12.08) | 0.144 |
| NA | NA | NA | NA |  |  | OTT | 1.00(1.00–1.00) | 0.047 |
| SICH | | | | | | | | |
| NIHSS | 1.08(1.03–1.15) | 0.005 | NIHSS | 1.08(1.02–1.13) | 0.007 | NA | NA | NA |
| NA | NA | NA | Recanalization | 0.30(0.14–0.67) | 0.003 | NA | NA | NA |

Abbreviations: OR, odds ratio; CI, confidence interval; mRS, modified Rankin Scale; SICH, symptomatic intracerebral hemorrhage; NIHSS, National Institutes of Health Stroke Scale; pc–ASPECTS. posterior circulation Alberta Stroke Program Early Computed Tomography Score; BATMAN, the Basilar Artery on Computed Tomography Angiography; OTT, onset to treatment time; PRT, puncture to recanalization time; IV, Intravenous thrombolysis; NA, not applicable;

**Table Ⅱ Multivariable Logistic Regression Model of Outcome Measures by PBT**

| ALL | Adjusted OR(95% CI) | *P* Value | EVT | Adjusted OR(95% CI) | *P* Value | SMT | Adjusted OR(95% CI) | *P* Value |
| --- | --- | --- | --- | --- | --- | --- | --- | --- |
| 3-month mRS 0-3 | | | | | | | | |
| NIHSS | 0.94(0.96–1.00) | <0.001 | NIHSS | 0.93(0.90–0.95) | <0.001 | NA | NA | NA |
| pc–ASPECTS | 1.67(1.42–1.97) | <0.001 | pc–ASPECTS | 1.67(1.41–1.97) | <0.001 | pc–ASPECTS | 1.82(1.01–3.26) | 0.045 |
| group | 4.12(1.94–8.73) | <0.001 | NA | NA | NA | NA | NA | NA |
| Occlusion site |  |  | Occlusion site |  |  | Occlusion site |  |  |
| Distal basilar artery | reference | 0.001 | Distal basilar artery | reference | 0.01 | Distal basilar artery | reference | 0.083 |
| Middle basilar artery | 0.26(0.14–0.48) | <0.001 | Middle basilar artery | 0.43(0.24–0.79) | 0.006 | Middle basilar artery | 0.07(0.01–0.54) | 0.01 |
| Proximal basilar artery | 0.52(0.25–1.07) | 0.075 | Proximal basilar artery | 0.71(0.36–1.41) | 0.33 | Proximal basilar artery | 0.52(0.04–6.27) | 0.61 |
| Vertebral artery–V4 segment | 0.37(0.17–0.77) | 0.008 | Vertebral artery–V4 segment | 0.37(0.19–0.75) | 0.005 | Vertebral artery–V4 segment | 0.48(0.04–5.92) | 0.569 |
| NA | NA | NA | PRT | 0.99(0.988–0.997) | 0.001 | NA | NA | NA |
| NA | NA | NA | Recanalization | 4.15(1.96–8.76) | 0.001 | NA | NA | NA |
| 3-month mortality | | | | | | | | |
| Age | 1.03(1.01–1.05) | 0.001 | Age | 1.03(1.01–1.04) | 0.013 | NA | NA | NA |
| NIHSS | 1.05(1.031–1.08) | <0.001 | NIHSS | 1.07(1.05–1.10) | <0.001 | NA | NA | NA |
| pc–ASPECTS | 0.78(0.70–0.88) | <0.001 | pc–ASPECTS | 0.75(0.66–0.86) | <0.001 | NA | NA | NA |
| BATMAN | 0.88(0.80–0.98) | 0.017 | PRT | 1.01(1.00–1.01) | <0.001 | BATMAN | 3.62(1.08–12.11) | 0.037 |
| group | 0.29(0.17–0.49) | <0.001 | Recanalization | 0.14(0.07–0.25) | <0.001 | NA | NA | NA |
| SBP | 1.01(1.00–1.02) | 0.025 | NA | NA | NA | NA | NA | NA |
| Occlusion site |  |  | NA | NA | NA | Occlusion site |  |  |
| Distal basilar artery | reference | 0.138 | NA | NA | NA | Distal basilar artery | reference | 0.222 |
| Middle basilar artery | 1.89(1.10–3.25) | 0.021 | NA | NA | NA | Middle basilar artery | 3.62(1.08–12.11) | 0.037 |
| Proximal basilar artery | 1.45(0.76–2.78) | 0.263 | NA | NA | NA | Proximal basilar artery | 2.81(0.39–20.26) | 0.306 |
| Vertebral artery–V4 segment | 11.66(0.88–3.13) | 0.116 | NA | NA | NA | Vertebral artery–V4 segment | 2.72(0.48–15.41) | 0.259 |
| In–hospital mortality 90d | | | | | | | | |
| Age | 1.02(1.00–1.04) | 0.026 | NA | NA | NA | pc–ASPECTS | 0.72(0.57–0.92) | 0.008 |
| NIHSS | 1.05(1.03–1.08) | <0.001 | NIHSS | 1.07(1.03–1.10) | <0.001 | NIHSS | 1.04(1.00–1.09) | 0.047 |
| pc–ASPECTS | 0.87(0.78–0.98) | 0.022 | BATMAN | 0.85(0.75–0.97) | 0.013 | Occlusion site |  |  |
| Neutrophils | 1.30(1.03–1.64) | 0.027 | PRT | 1.00(1.00–1.01) | 0.015 | Distal basilar artery | reference | 0.108 |
| group | 0.45(0.28–0.74) | 0.002 | Recanalization | 0.42(0.25–0.71) | 0.001 | Middle basilar artery | 3.92(1.26–12.26) | 0.019 |
| diabetes mellitus | 1.72(1.09–2.72) | 0.02 | NA | NA | NA | Proximal basilar artery | 1.86(0.30–11.61) | 0.505 |
| NA | NA | NA | NA | NA | NA | Vertebral artery–V4 segment | 3.76(0.83–16.91) | 0.085 |
| SICH | | | | | | | | |
| NIHSS | 1.08(1.02–1.14) | 0.011 | NIHSS | 1.07(1.01–1.13) | 0.017 | NA | NA | NA |
| group | 8.60(1.09–67.88) | 0.041 | Recanalization | 0.33(0.15–0.75) | 0.008 | NA | NA | NA |

Abbreviations: OR, odds ratio; CI, confidence interval; mRS, modified Rankin Scale; SICH, symptomatic intracerebral hemorrhage; NIHSS, National Institutes of Health Stroke Scale; pc–ASPECTS. posterior circulation Alberta Stroke Program Early Computed Tomography Score; BATMAN, the Basilar Artery on Computed Tomography Angiography; OTT, onset to treatment time; PRT, puncture to recanalization time; IV, Intravenous thrombolysis; NA, not applicable;

**Table Ⅲ Multivariable Logistic Regression Model of Outcome Measures by MBT**

| ALL | Adjusted OR(95% CI) | *P* Value | EVT | Adjusted OR(95% CI) | *P* Value | SMT | Adjusted OR(95% CI) | *P* Value |
| --- | --- | --- | --- | --- | --- | --- | --- | --- |
| 3-month mRS 0-3 | | | | | | | | |
| NIHSS | 0.94(0.91–0.96) | <0.001 | NIHSS | 0.92(0.90–0.95) | <0.001 | NIHSS | 0.91(0.84–0.99) | 0.024 |
| pc–ASPECTS | 1.70(1.45–2.00) | <0.001 | pc–ASPECTS | 1.70(1.44–2.01) | <0.001 | NA | NA | NA |
| group | 4.49(2.13–9.48) | <0.001 | NA | NA | NA | NA | NA | NA |
| Occlusion site |  |  | Occlusion site |  |  | Occlusion site |  |  |
| Distal basilar artery | reference | <0.001 | Distal basilar artery | reference | 0.009 | Distal basilar artery | reference | 0.081 |
| Middle basilar artery | 0.26(0.14–0.48) | <0.001 | Middle basilar artery | 0.42(0.23–0.77) | 0.005 | Middle basilar artery | 0.07(0.01–0.56) | 0.012 |
| Proximal basilar artery | 0.51(0.25–1.05) | 0.068 | Proximal basilar artery | 0.68(0.34–1.33) | 0.254 | Proximal basilar artery | 0.76(0.07–8.66) | 0.828 |
| Vertebral artery–V4 segment | 0.37(0.18–0.78) | 0.009 | Vertebral artery–V4 segment | 0.37(0.18–0.74) | 0.005 | Vertebral artery–V4 segment | 0.50(0.04–6.57) | 0.6 |
| NA | NA | NA | PRT | 0.99(0.988–0.997) | 0.001 | IV | 10.45(1.27–86.08) | 0.029 |
| NA | NA | NA | Recanalization | 3.96(1.90–8.27) | <0.001 | NA | NA | NA |
| 3-month mortality | | | | | | | | |
| Age | 1.03(1.01–1.05) | 0.003 | Age | 1.02(1.00–1.04) | 0.024 | NIHSS | 1.06(1.01–1.11) | 0.015 |
| NIHSS | 1.06(1.04–1.08) | <0.001 | NIHSS | 1.08(1.05–1.11) | <0.001 | BATMAN | 0.65(0.50–0.88) | 0.001 |
| pc–ASPECTS | 0.77(0.69–0.86) | <0.001 | pc–ASPECTS | 0.74(0.65–0.84) | <0.001 | NA | NA | NA |
| BATMAN | 0.88(0.809–0.98) | 0.016 | PRT | 1.01(1.00–1.01) | <0.001 | NA | NA | NA |
| group | 0.28(0.17–0.46) | <0.001 | Recanalization | 0.14(0.07–0.25) | <0.001 | NA | NA | NA |
| SBP | 1.01(1.00–1.02) | 0.021 | NA | NA | NA | NA | NA | NA |
| Occlusion site |  |  | NA | NA | NA | NA | NA | NA |
| Distal basilar artery | reference | 0.133 | NA | NA | NA | NA | NA | NA |
| Middle basilar artery | 1.90(1.11–3.25) | 0.019 | NA | NA | NA | NA | NA | NA |
| Proximal basilar artery | 1.50(0.79–2.86) | 0.216 | NA | NA | NA | NA | NA | NA |
| Vertebral artery–V4 segment | 1.63(0.87–3.05) | 0.132 | NA | NA | NA | NA | NA | NA |
| In–hospital mortality 90d | | | | | | | | |
| Age | 1.02(1.00–1.04) | 0.033 | NIHSS | 1.07 (1.04–1.106) | <0.001 | NIHSS | 1.05(1.01–1.09) | 0.017 |
| NIHSS | 1.06(1.03–1.08) | <0.001 | BATMAN | 0.85(0.75–0.97) | 0.013 | pc–ASPECTS | 0.75(0.59–0.95) | 0.017 |
| pc–ASPECTS | 0.87(0.77–0.97) | 0.016 | PRT | 1.00(1.00–1.01) | 0.014 | Occlusion site |  |  |
| Neutrophils | 1.30(1.03–1.63) | 0.028 | Recanalization | 0.42(0.25–0.71) | 0.001 | Distal basilar artery | reference | 0.165 |
| group | 0.45(0.27–0.73) | 0.001 | NA | NA | NA | Middle basilar artery | 3.29(1.09–9.89) | 0.034 |
| Diabetes mellitus | 1.75(1.11–2.76) | 0.016 | NA | NA | NA | Proximal basilar artery | 1.54(0.25–9.58) | 0.642 |
| NA | NA | NA | NA | NA | NA | Vertebral artery–V4 segment | 3.17(0.74–13.70) | 0.122 |
| SICH | | | | | | | | |
| NIHSS | 1.08(1.02–1.15) | 0.007 | NIHSS | 1.074(1.018–1.133) | 0.009 | NA | NA | NA |
| NA | NA | NA | Recanalization | 0.32(0.14–0.70) | 0.005 | NA | NA | NA |

Abbreviations: OR, odds ratio; CI, confidence interval; mRS, modified Rankin Scale; SICH, symptomatic intracerebral hemorrhage; NIHSS, National Institutes of Health Stroke Scale; pc–ASPECTS. posterior circulation Alberta Stroke Program Early Computed Tomography Score; BATMAN, the Basilar Artery on Computed Tomography Angiography; OTT, onset to treatment time; PRT, puncture to recanalization time; IV, Intravenous thrombolysis; NA, not applicable;

**Table Ⅳ Correlation of body temperature with other variables**

|  | | ABT | PBT | MBT | WBC | Neutrophil | Lymphocyte | CRP | PCT | ODT | ORT | LOS |
| --- | --- | --- | --- | --- | --- | --- | --- | --- | --- | --- | --- | --- |
| ABT | r | 1.000 | .257^**^ | –.259^**^ | .153^**^ | .151^**^ | –0.059 | 0.044 | 0.115 | .179^**^ | .214^**^ | –0.058 |
|  | *P* |  | 0.000 | 0.000 | 0.000 | 0.000 | 0.147 | 0.470 | 0.060 | 0.000 | 0.000 | 0.134 |
| PBT | r | .257^**^ | 1.000 | .802^**^ | .122^**^ | .125^**^ | –0.039 | .126^*^ | 0.089 | –0.006 | .126^**^ | –.083^*^ |
|  | *P* | 0.000 |  | 0.000 | 0.002 | 0.002 | 0.336 | 0.037 | 0.145 | 0.897 | 0.004 | 0.033 |
| MBT | r | –.259^**^ | .802^**^ | 1.000 | 0.025 | 0.027 | –0.019 | 0.071 | 0.015 | –.117^**^ | 0.011 | –0.037 |
|  | *P* | 0.000 | 0.000 |  | 0.526 | 0.508 | 0.646 | 0.240 | 0.801 | 0.006 | 0.810 | 0.344 |
| WBC | r | .153^**^ | .122^**^ | 0.025 | 1.000 | .964^**^ | 0.028 | .150^*^ | –0.031 | 0.062 | .119^**^ | –.157^**^ |
|  | *P* | 0.000 | 0.002 | 0.526 |  | 0.000 | 0.499 | 0.014 | 0.616 | 0.154 | 0.008 | 0.000 |
| Neutrophil | r | .151^**^ | .125^**^ | 0.027 | .964^**^ | 1.000 | –.173^**^ | .144^*^ | –0.005 | .095^*^ | .146^**^ | –.164^**^ |
|  | *P* | 0.000 | 0.002 | 0.508 | 0.000 |  | 0.000 | 0.019 | 0.941 | 0.031 | 0.001 | 0.000 |
| Lymphocyte | r | –0.059 | –0.039 | –0.019 | 0.028 | –.173^**^ | 1.000 | –0.088 | –0.115 | –.102^*^ | –.127^**^ | 0.050 |
|  | *P* | 0.147 | 0.336 | 0.646 | 0.499 | 0.000 |  | 0.150 | 0.069 | 0.023 | 0.006 | 0.219 |
| CRP | r | 0.044 | .126^*^ | 0.071 | .150^*^ | .144^*^ | –0.088 | 1.000 | 0.112 | 0.033 | 0.094 | –0.100 |
|  | *P* | 0.470 | 0.037 | 0.240 | 0.014 | 0.019 | 0.150 |  | 0.191 | 0.626 | 0.176 | 0.100 |
| PCT | r | 0.115 | 0.089 | 0.015 | –0.031 | –0.005 | –0.115 | 0.112 | 1.000 | 0.070 | 0.032 | –0.047 |
|  | *P* | 0.060 | 0.145 | 0.801 | 0.616 | 0.941 | 0.069 | 0.191 |  | 0.293 | 0.650 | 0.444 |
| ODT | r | .179^**^ | –0.006 | –.117^**^ | 0.062 | .095^*^ | –.102^*^ | 0.033 | 0.070 | 1.000 | .770^**^ | –0.050 |
|  | *P* | 0.000 | 0.897 | 0.006 | 0.154 | 0.031 | 0.023 | 0.626 | 0.293 |  | 0.000 | 0.241 |
| ORT | r | .214^**^ | .126^**^ | 0.011 | .119^**^ | .146^**^ | –.127^**^ | 0.094 | 0.032 | .770^**^ | 1.000 | –0.085 |
|  | *P* | 0.000 | 0.004 | 0.810 | 0.008 | 0.001 | 0.006 | 0.176 | 0.650 | 0.000 |  | 0.055 |
| LOS | r | –0.058 | –.083^*^ | –0.037 | –.157^**^ | –.164^**^ | 0.050 | –0.100 | –0.047 | –0.050 | –0.085 | 1.000 |
|  | *P* | 0.134 | 0.033 | 0.344 | 0.000 | 0.000 | 0.219 | 0.100 | 0.444 | 0.241 | 0.055 |  |

Abbreviations: ABT, admission body temperature; PBT, peak body temperature; MBT, minus of body temperature; WBC, white blood cell; CRP, C–reactive protein; PCT, procalcitonin; ODT, onset to door time; ORT, onset to recanalization time; LOS, length of stay; *. At the 0.05 level (two–tailed), the results are significant **. At the 0.01 level (two–tailed), the results are significant.
